# Fitting of 2D WAXD data: Mesophases in polymer fibers

**DOI:** 10.1016/j.dib.2021.107466

**Published:** 2021-10-09

**Authors:** Edith Perret, Rudolf Hufenus

**Affiliations:** a Laboratory for Advanced Fibers, Empa, Swiss Federal Laboratories for Materials Science and Technology, Lerchenfeldstrasse 5, St. Gallen 9014, Switzerland; b Center for X-ray Analytics, Empa, Swiss Federal Laboratories for Materials Science and Technology, Überlandstrasse 129, Dübendorf 8600, Switzerland

**Keywords:** X-ray diffraction, Mesophases, WAXD fitting algorithms

## Abstract

This data article presents fitting results of wide-angle x-ray diffraction (WAXD) patterns of melt-spun polymer fibers from amorphous materials (polycarbonate (PC), cyclo-olefin polymer (COP), copolyamide (coPA), polyethylene terephthalate glycol (PETG)) and semi-crystalline materials (polyethylene terephthalate (PET), poly-3-hydroxybutyrate(P3HB)). The data was fit using the fitting algorithms, previously described in the publication by Perret and Hufenus ‘Insights into strain-induced solid mesophases in melt-spun polymer fibers’ [1]. Fitting results of WAXD data and details about azimuthal, equatorial, meridional or off-axis profiles are presented in sections 1.1-1.2. SAXS patterns of fibers, melt-spun from amorphous materials, are shown in section 1.3. Fiber production parameters are given in section 2.1, and a description of the WAXD measurements and fitting details, e.g., the chosen fitting parameters, are given in section 2.2.

## Specifications Table

**Table:** 

Subject	Materials Science: Polymers and Plastics
Specific subject area	Structure of melt-spun polymer filaments.
Type of data	Table Image Figure Equations
How data were acquired	**Instruments:** X-ray data (WAXD/SAXS): Bruker Nanostar U diffractometer (Bruker AXS, Karlsruhe, Germany) **Software:** DIFFRAC.EVA (version 4.2., Bruker AXS, Karlsruhe, Germany), Python codes
Data format	Raw Analyzed
Parameters for data collection	WAXD and SAXS patterns of all filaments were recorded on a Bruker Nanostar U diffractometer (Bruker AXS, Karlsruhe, Germany) with Cu-Kα radiation (λ = 1.5419 Å), a VÅNTEC-2000 MikroGap area detector and a 300 µm wide beam defining pinhole.
Description of data collection	WAXD patterns have been acquired from drawn melt-spun fibers from either amorphous or semi-crystalline materials. The WAXD patterns have subsequently been fitted in order to obtain structural information about the amorphous phase, mesophase and crystalline phases present in the fibers. SAXS patterns have been measured in order to verify that no lamellae are present in the fibers.
Data source location	Empa, St. Gallen, Switzerland
Data accessibility	Mendeley Data DOI: http://dx.doi.org/10.17632/w73svj4xwr.3 http://dx.doi.org/10.17632/w73svj4xwr.3
Related research article	E. Perret, R. Hufenus Insights into strain-induced solid mesophases in melt-spun polymer fibers Polymer DOI: https://doi.org/10.1016/j.polymer.2021.124010

## Value of the Data

• The presented WAXD data highlights the presence of mesophases in many drawn melt-spun polymer fibers.
• The fitting results of 2D WAXD data are of potential interest to the polymer fiber community.
• Details about the fitting procedure are of potential interest to other researchers.
• SAXS pattern confirm that the mesophases are non-crystalline.

## 1 Data Description

### 1.1 WAXD data: drawn fibers melt-spun from amorphous polymers

#### 1.1.1 Cyclo-olefin polymer monofilaments

**Fig. 1:**
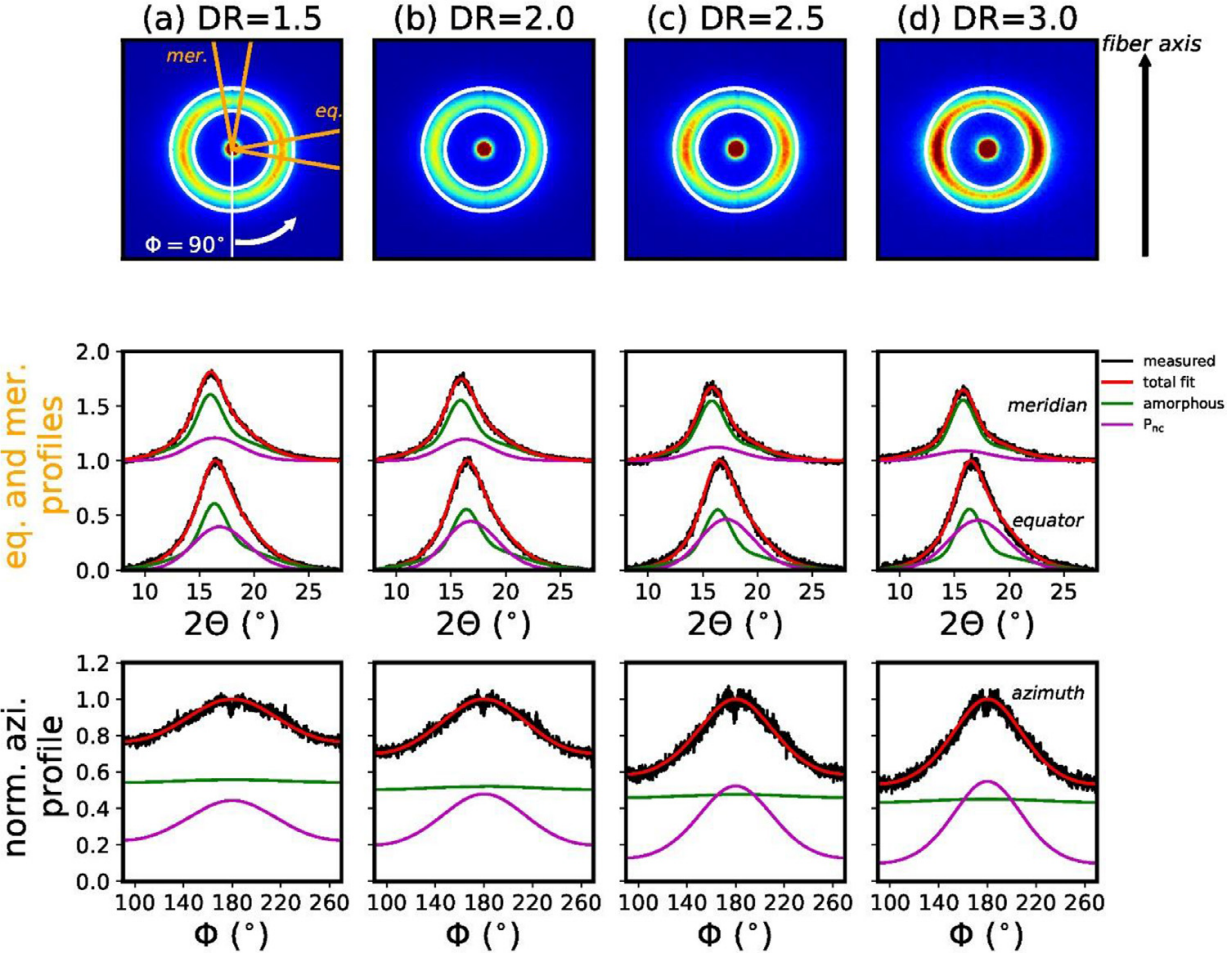
Measured WAXD patterns of COP fibers (top row) with corresponding fits of equatorial (eq.), meridional (mer.) (middle row) and azimuthal profiles (bottom row). The equatorial and meridional profiles have been normalized to the equatorial peak intensities and the meridional profiles are offset by +1 for better visibility. The integrated meridional (mer.) and equatorial (eq.) sectors (opening angle 20°) are highlighted with orange lines and the integrated azimuthal ring is shown with white circles.

Fig. 1 shows the measured WAXD patterns for all fibers, including the fits of the meridional, equatorial and azimuthal profiles.

#### 1.1.2 Co-polyamide monofilaments

**Fig. 2:**
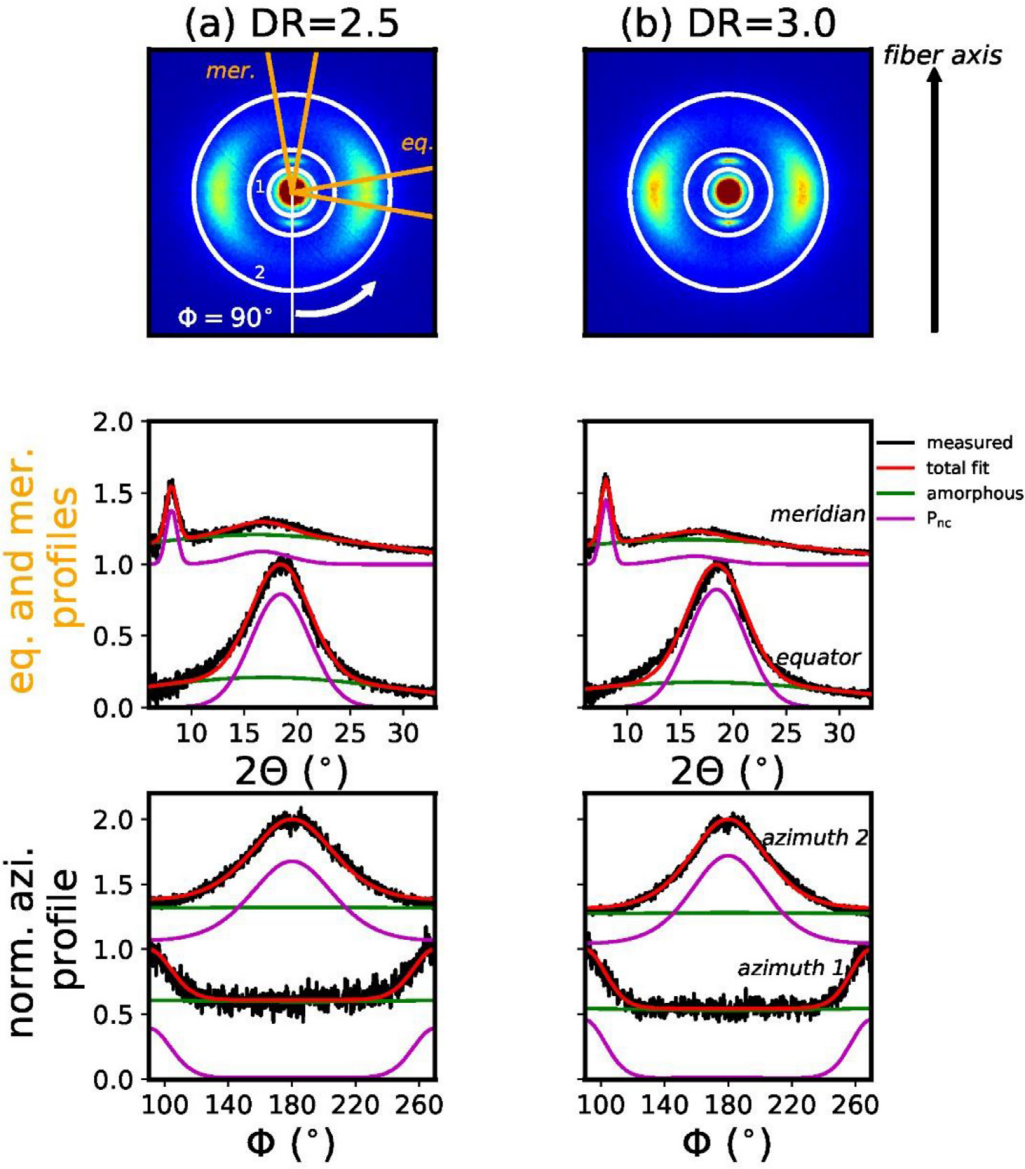
Measured WAXD patterns of coPA fibers (top row) with corresponding fits of equatorial (eq.), meridional (mer.) (middle row) and two azimuthal profiles (bottom row). The integrated meridional (mer.) and equatorial (eq.) sectors (opening angle 20°) are highlighted with orange lines and the two integrated azimuthal rings are shown with white circles.

Fig. 2 shows the measured WAXD patterns for all coPA fibers, including the fits of the meridional, equatorial and azimuthal profiles.

#### 1.1.3 PETG/PMP bicomponent monofilament

**Fig. 3:**
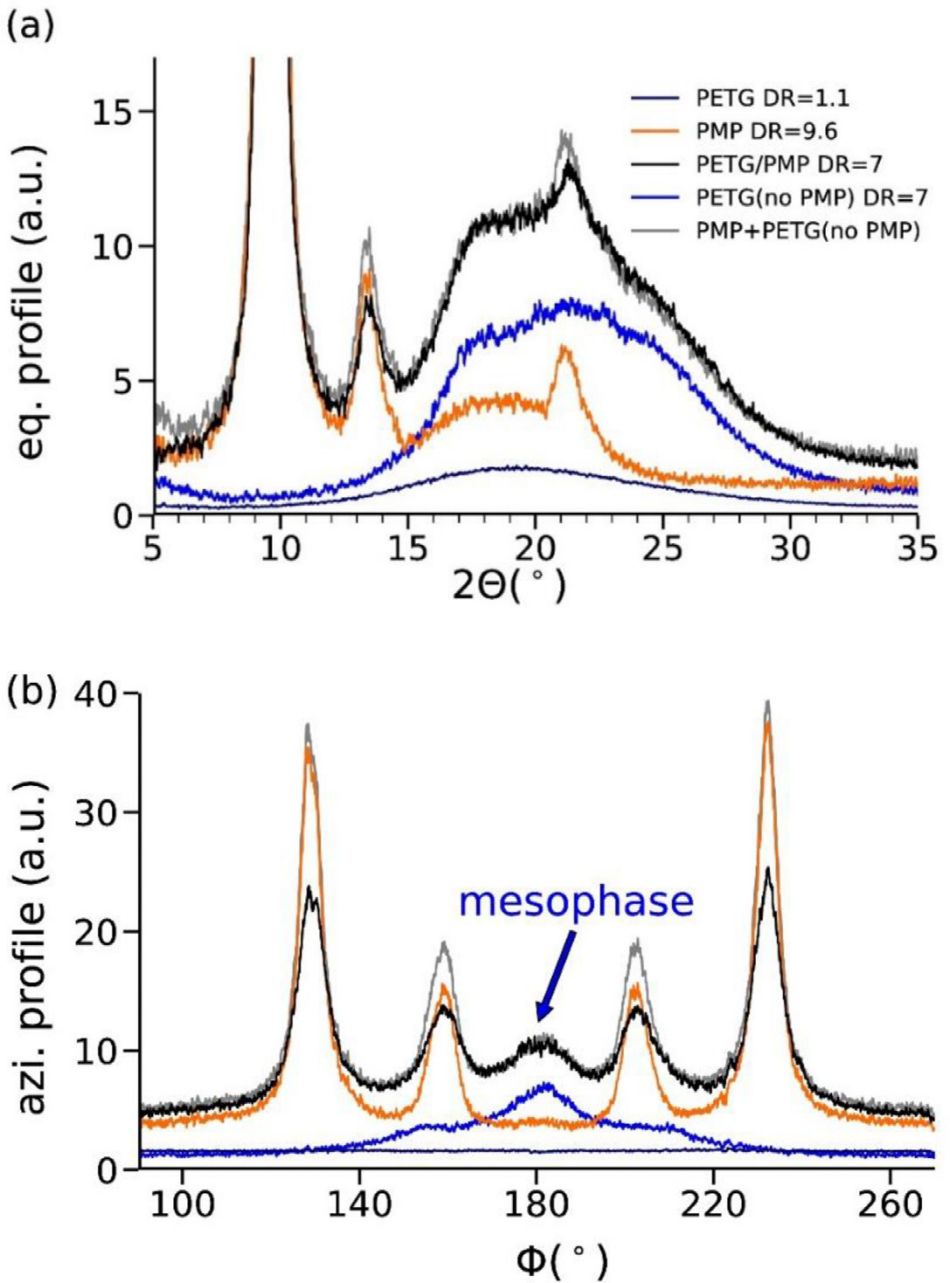
(a) Equatorial and (b) azimuthal profiles of PETG and PMP monofilaments, of a PETG/PMP bicomponent fiber, and of the PETG core. Additionally, the sum of the profiles from the PMP filament and the PETG core is shown in grey.

Extracted equatorial profiles (Fig. 3a) and azimuthal profiles (Fig. 3b) highlight the existence of a mesophase in the PETG core material. While the as-spun PETG monofilament with DR = 1.1 shows an amorphous ring (dark blue curves), the PMP shows an overlap of a highly oriented crystalline phase with an amorphous ring (orange curves). The equatorial profile of the bicomponent fiber (black curves, Fig. 3a) shows a strong increase of the intensity in the region between 15 and 28°. Removal of the PMP sheath shows that this increase in intensity is attributed to a mesophase in the highly drawn PETG core (light blue curves). A summation of the profiles from the highly drawn PMP (orange curves) monofilament and the PETG core (dark blue curves) leads to profiles (grey curves) which are very similar to the measured profiles from the bicomponent fiber (black curves). This proves that the PETG core structure was not impacted by the dissolution of the PMP sheath.

### 1.2 WAXD data: drawn fibers melt-spun from semi-crystalline polymers

#### 1.2.1 Polyethylene terephthalate monofilament

Table 1 summarizes the best fit parameters for the mesophase and crystalline phase in the PET monofilament.

**Table 1:** Best fit parameters for the mesophase and crystalline phase in PET monofilament.

Amorphous				Crystalline phase		
*A*_norm_	$\mu_{1}$(°)	$d_{1}$(Å)	$\sigma_{1}$(°)	*p*_off-axis_	*w*_off-axis_ (Å^−1^)	*W*_off-axis_ (nm)
0.26	20.0	4.4	11.6	392	0.0700	1.4

#### 1.2.2 Poly-3-hydroxybutyrate monofilaments

Some best fit crystalline parameters are summarized in Table 2, and the other parameters of the equatorial reflections can be found in the publication by Perret and Hufenus [1]. Looking at the fitting results of the crystalline phase, it is noticed that some of the parameters could also be fixed in order to reduce the number of fitting parameters, since they do not change as a function of the applied stress (*e.g.* Δx_12_=0.00, Δx_3_=0.03, *f* = 0.7, p_0_ = 0.4).

**Table 2:** A selection of fitting parameters of crystalline phase, amorphous phase, equatorial streak and background.

	Crystalline		Amorphous						Streak and background				
P3HB fibers (stress)	*p*_off-axis_	*w*_off-axis_	*A*_norm_	*B*_norm_	$\mu_{1}$(°)	$\mu_{2}$(°)	$\sigma_{1}$(°)	$\sigma_{2}$(°)	*C*_norm_	$\mu_{1}$(°)	$\sigma_{1}$(°)	Δ*X*_st_	*bkg*
0 MPa	200	0.0143	0.24	0.12	22.0	18.9	0.8	2.9	0.24	16.5	4.7	4.1	0.11
128 MPa	165	0.0148	0.25	0.12	22.1	19.0	0.7	3.0	0.25	17.2	4.7	4.4	0.13
168 MPa	154	0.0160	0.26	0.15	22.1	18.8	0.7	2.9	0.27	16.9	4.7	4.5	0.17

**Fig. 4:**
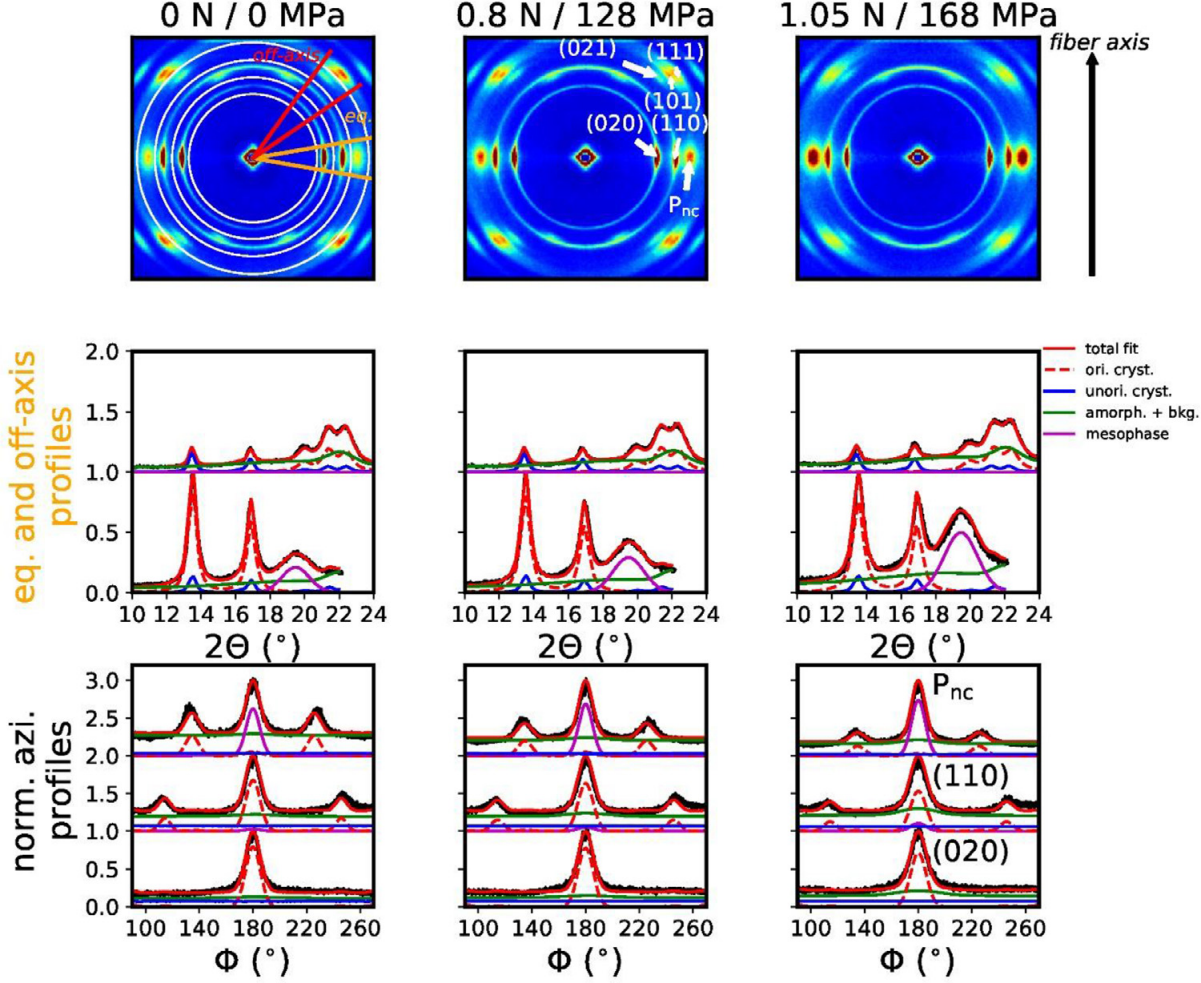
Measured WAXD patterns of P3HB fibers (top row) with corresponding fits of equatorial (eq.), off-axis profiles (middle row) and normalized azimuthal profiles (bottom row). The equatorial and off-axis profiles have been normalized to the equatorial (020) peak intensity, and the off-axis profiles are offset by +1 for better visibility. The integrated off-axis sector lies between the red lines, and the equatorial sector between orange lines. Integrated azimuthal rings are shown with white circles.

Fig. 4 shows the profile fitting results.

### 1.3 SAXS data: drawn fibers melt-spun from amorphous polymers

#### 1.3.1 Polycarbonate monofilaments

**Fig. 5:**
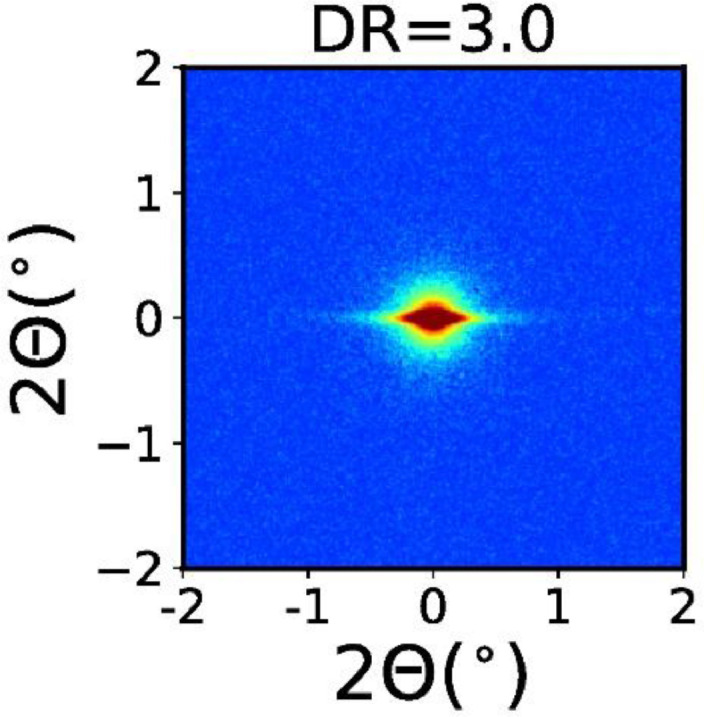
SAXS pattern of drawn PC fiber with DR=3.

The SAXS pattern of drawn PC monofilament bundles (DR = 3) is shown in Fig. 5. Note that all other SAXS patterns in the other sections show the same angular range.

#### 1.3.2 Co-polyamide monofilaments

**Fig. 6:**
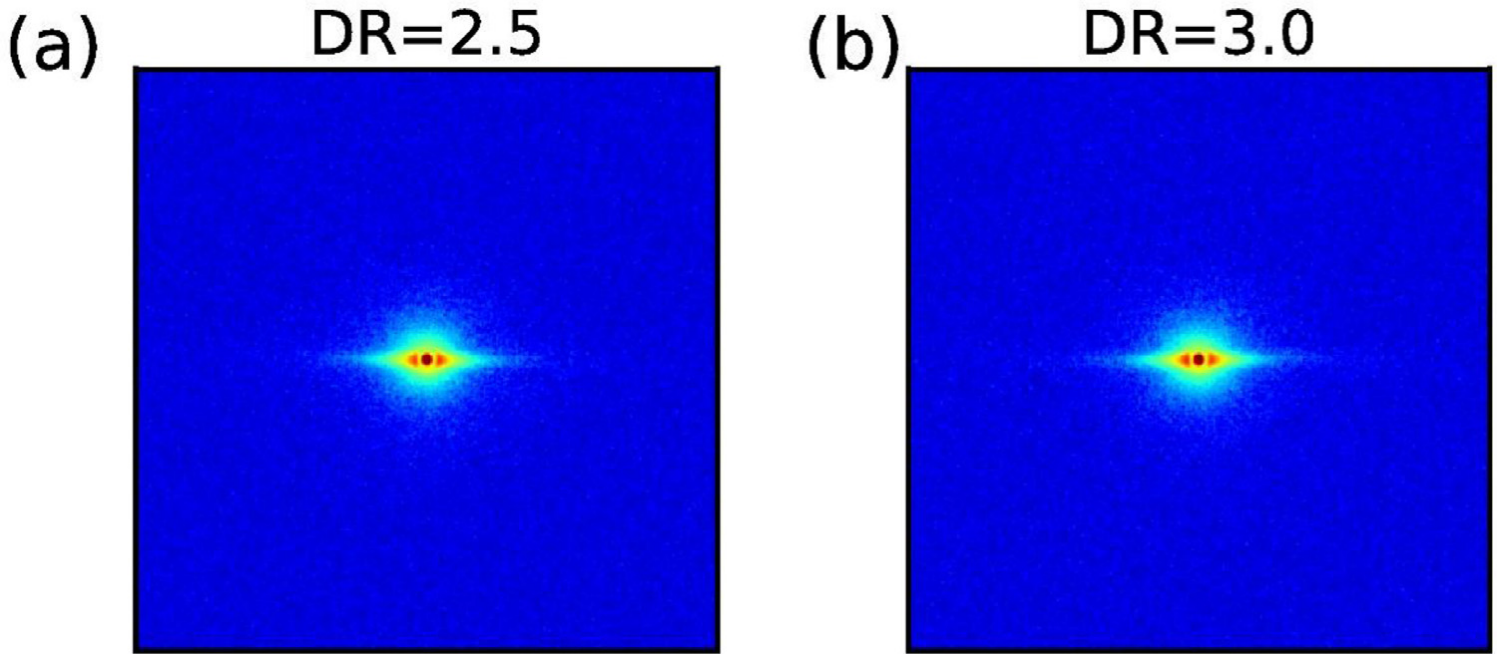
SAXS patterns of drawn coPA monofilament bundles.

The SAXS pattern of the drawn coPA monofilament bundles are shown in Fig. 6.

#### 1.3.3 PETG/PMP bicomponent monofilament

**Fig. 7:**
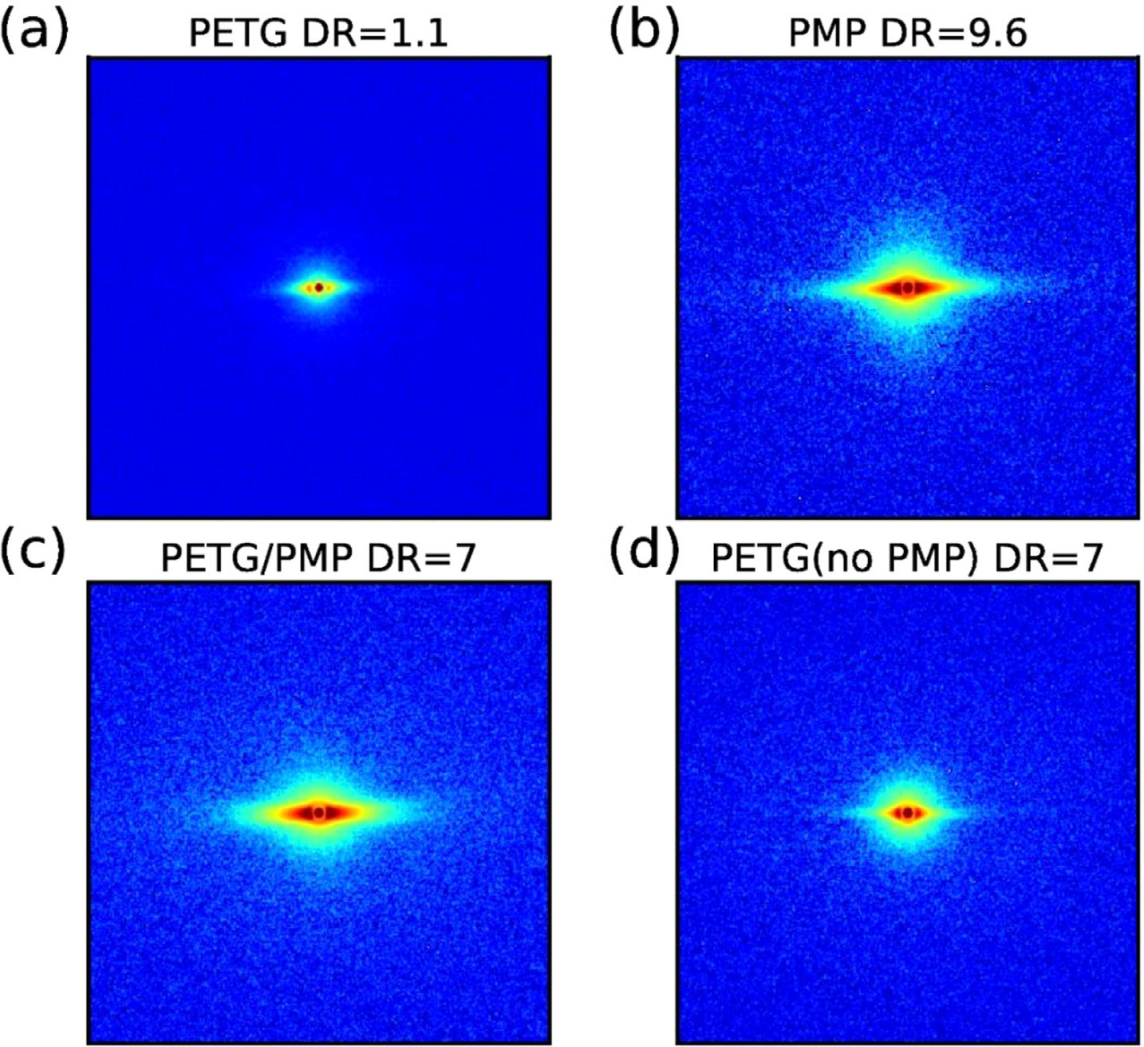
Measured SAXS patterns of (a) PETG monofilament, (b) PMP monofilament, (c) PETG/PMP bicomponent fiber, (d) PETG core of bicomponent fiber with removed sheath.

The SAXS pattern of the PETG and PMP monofilament bundles, as well as of the bicomponent PETG/PMP fiber bundles and the PETG core bundles, are shown in Fig. 7.

The electron density difference between crystalline and amorphous phases is very small for PMP at room temperature. Normally, in most polymers, the crystalline phase is more dense than the amorphous phase. Only staining with iodine or heating/stretching could make the lamellar peaks of PMP visible in SAXS [2].

## 2 Experimental Design, Materials and Methods

The materials, experimental methods for WAXD and SAXS measurements, as well as fitting methods for WAXD patterns, have been previously described in detail in the article by Perret and Hufenus [1]. Text, that has been put between quotes, has been taken from said article.

### 2.1 Fiber production parameters

Details about raw materials, fiber types, fiber diameters, fineness and draw ratios are given in the publication by Perret and Hufenus [1]. Typically, the melting of the polymers has been performed with a single-screw extruder with a diameter of 18 mm and a length of 450 mm. In case another extruder was used, it is specifically indicated in the descriptions below.

#### 2.1.1 Polycarbonate monofilaments

Table 3 summarizes the production parameters of melt-spun PC monofilaments. The filaments have been melt-spun through the core capillary of a “multiple” bicomponent spinneret die [3,4]. The core capillary had an inner diameter of 0.8 mm and an outer diameter of 1.2 mm.

**Table 3:** Production parameters of melt-spun PC monofilaments.

Fiber no.	Draw ratio	spin pack (°C)	spin press. (bar)	air cool. temp. (°C)	godet 1 speed/temp. (m/min,°C)	godet 2 speed/temp. (m/min,°C)	godet 3 speed/temp. (m/min,°C)	godet 4 speed/temp. (m/min,°C)	godet 5 speed/temp. (m/min,°C)	winder speed (m/min)
1929	1	295	121	13	300/70	300/50	300/40	300/40	300/30	300
1932	2	295	123	10	300/85	600/60	600/40	600/40	600/30	600
1931	3	295	123	10	300/85	900/60	900/40	900/40	900/30	900

#### 2.1.2 Cyclo-olefin polymer monofilaments

Table 4 summarizes the production parameters of melt-spun COP monofilaments. The filaments have been melt-spun through the core capillary of a "multiple" bicomponent spinneret die [3,4]. The core capillary had an inner diameter of 0.4 and an outer diameter of 0.7 mm.

**Table 4:** Production parameters of melt-spun COP monofilaments.

Fiber no.	Draw ratio	spin pack (°C)	spin press. (bar)	air cool. temp. (°C)	godet 1 speed/temp. (m/min,°C)	godet 2 speed/temp. (m/min,°C)	godet 3 speed/temp. (m/min,°C)	winder speed (m/min)
807	1.5	250	20.5	19	200/100	290/140	300/80	300
806	2.0	250	21.2	19	200/100	290/140	400/80	400
808	2.5	250	21.2	19	200/100	490/140	500/80	500
809	3.0	250	21.2	19	200/100	590/140	600/80	600

#### 2.1.3 Co-polyamide monofilaments

Table 5 summarizes the production parameters of melt-spun coPA monofilaments. The filament with a draw ratio of 2.5 has been melt-spun through a capillary die with a diameter of 0.5 mm and a length of 2 mm. The filament with a draw ratio of 3.0 was melt-spun through the core of a "multiple" bicomponent spinneret die [3,4], which had a capillary with an inner diameter of 0.6 mm and an outer diameter of 0.9 mm.

**Table 5:** Production parameters of melt-spun coPA monofilaments.

Fiber no.	Draw ratio	spin pack (°C)	spin press. (bar)	air cool. temp. (°C)	godet 1 speed/temp. (m/min,°C)	godet 2 speed/temp. (m/min,°C)	godet 3 speed/temp. (m/min,°C)	winder speed (m/min)
1007	2.5	235	1	9	400/90	990/120	1000/60	1000
1083	3.0	255	160	10	300/120	890/90	900/50	900

#### 2.1.4 PETG, PMP monofilaments and PETG/PMP bicomponent monofilament

Table 6 summarizes the production parameters of melt-spun PETG monofilaments. A single-screw extruder with a diameter of 13 mm and a length of 325 mm was used to melt the PETG polymer. Another single-screw extruder with a diameter of 18 mm and a length of 450 mm was used to melt the PMP polymer. The filaments have been melt-spun with a "single" bicomponent spinneret die [3,4] with an injector with bore diameter of 1.9 mm, and a die capillary with diameter of 1.5 mm and length of 5 mm.

**Table 6:** Production parameters of melt-spun PETG and PMP monofilaments as well as of a core/sheath bicomponent fiber.

	Fiber no.	Draw ratio	spin pack (°C)	spin press. (bar)	air cool. temp. (°C)	godet 1 speed/temp. (m/min,°C)	godet 2 speed/temp. (m/min,°C)	godet 3 speed/temp. (m/min,°C)	godet 4 peed/temp. (m/min,°C)	godet 5 speed/temp. (m/min,°C)	winder speed (m/min)
Monofil.	1702 PETG	1.1	240	35	11	-	-	140/40	145/40	150/40	150
Monofil.	1706 PMP	9.6	250	21.8	98	50/115	480/80	480/35	480/35	480/30	470

Bicomp. core	1707 PETG	7.0	250	9	98	50/100	350/80	350/35	350/35	350/30	350
Bicomp. sheath	1707 PMP	7.0	250	23	98	50/100	350/80	350/35	350/35	350/30	350

#### 2.1.5 Poly-3-hydroxybutyrate monofilament

A single-screw extruder with a diameter of 13 mm and a length of 325 mm was used to melt the P3HB polymer [5–8]. The filaments have been melt-spun through a capillary die with a diameter of 0.5 mm and a length of 2 mm. The production parameters are given in Table 7.

**Table 7:** Production parameters of melt-spun P3HB monofilament.

Fiber no.	Draw ratio	spin pack (°C)	spin press. (bar)	air cool. temp. (°C)	godet 1 speed/temp. (m/min,°C)	godet 2 speed/temp. (m/min,°C)	godet 3 speed/temp. (m/min,°C)	winder speed (m/min)
1108	6.0	150	50	10	360/90	360/100	360/40	360

### 2.2 WAXD/SAXS

'A Bruker Nanostar U diffractometer (Bruker AXS, Karlsruhe, Germany) was used to measure the WAXD and small-angle x-ray scattering (SAXS) patterns of melt-spun filaments or filament bundles. The Cu Kα radiation (*λ* = 1.5419 Å) was sent through a beam defining pinhole of 300 µm to the filaments, and the diffraction pattern was recorded with a VÅNTEC-2000 MikroGap area detector (Bruker AXS, Karlsruhe, Germany). The distance of the sample to the active detector area was typically close to 9.2 cm, and the exposure times were either 1800 s or 3600 s for WAXD. The measured intensities of WAXD patterns have been multiplied with the draw ratio of the fibers, in order to account for the thinning of the fibers due to drawing. WAXD measurements were performed in order to quantify the mesophase content and obtain structural information. SAXS measurements were performed to verify if lamellae are present in the fibers. For SAXS, the sample to active detector area distance was typically close to 110.5 cm, and exposure times were 4600s. ' Note that all Bruker (.gfrm) images from the Mendeley repository can all be plotted with the open source Fabio python package (https://pypi.org/project/fabio/).

The focus of the study was on determining structural details about the higly oriented non-crystalline mesophase, P_nc_ [8]. The mesophase consists of highly oriented, conformationally disordered macromolecules [[5], [6], [7], [8], [9], [10], [11], [12]].

#### 2.2.1 WAXD analysis: hermans' orientation parameter applied to mesophases

The Hermans' orientation parameter [13] of the mesophase can be calculated from the azimuthal profile of the mesophase using the following equation:

(1) $$f_{\mathrm{Pnc}}=\frac{3\langle \cos^{2}\phi \rangle -1}{2}$$

where $\langle \cos^{2}\phi \rangle$ reflects the azimuthal spread of the mesophase. Here, the azimuthal angle is zero at the maximum of the mesophase. Assuming rotational symmetry around the fiber axis, the term $\langle \cos^{2}\phi \rangle$ is given by

(2) $$\langle \cos^{2}\phi \rangle =\frac{\int_{0}^{\pi /2}I\left(\phi \right)\cos^{2}\phi \sin\phi \;d\phi }{\int_{0}^{\pi /2}I\left(\phi \right)\sin\phi \;d\phi }$$

where I(ϕ) is the azimuthal profile of the highly oriented part of the mesophase. If $f_{\text{Pnc}}=1$, then the chains of the mesophase are completely aligned parallel to the fiber axis. If $f_{\text{Pnc}}=0$, then the chains are randomly oriented.

#### 2.2.2 WAXD fitting parameters

##### 2.2.2.1 Polycarbonate monofilaments

For the as-spun fiber, 6 fitting parameters were used for two amorphous rings (heights, positions and widths). For the drawn PC fibers, 15 fitting parameters were used. 5 fitting parameters resulted from the mesophase (*D,*$\;\mu_{\text{Pnc}},$ $\sigma_{\text{Pnc}}$*,* $\Delta X_{\text{Pnc}}$, *a*) and 10 from three elliptic amorphous contributions (*A, B, C*, $\mu_{1},\;\mu_{2}$, $\mu_{3}$, $\sigma_{1},\sigma_{2}$, $\sigma_{3},$ *k*).

##### 2.2.2.2 Cyclo-olefin polymer monofilaments

The fitting procedure for the drawn COP fibers amounted to 12 fitting parameters [1]. 5 fitting parameters were used for the mesophase and 7 fitting parameters for two elliptic amorphous contributions (*A, B*, $\mu_{1},\;\mu_{2}$, $\sigma_{1},\sigma_{2}$, *k*).

##### 2.2.2.3 Co-polyamide monofilaments

The drawn coPA fibers were fitted with 13 fitting parameters consisting of 5 fitting parameters for the equatorial mesophase peak (*D,*$\;\mu_{\text{Pnc}},$ $\sigma_{\text{Pnc}}$*, a,* $\Delta X_{\text{Pnc}}$), 4 fitting parameters for the meridional mesophase peak (*E,*$\;\mu_{\text{mer}},$ $\sigma_{\text{mer}}$*,* $\Delta X_{\text{mer}}$) and 4 fitting parameters for the elliptic amorphous phase (*A*, $\mu_{1}$, $\sigma_{1}$, *k*).

##### 2.2.2.4 Polyethylene terephthalate monofilament

For the fits of the drawn PET filament, we have used in total 17 fitting parameters [1]. 9 fitting parameters were used for the crystalline part (*p, p*_off-axis_, *p*_0_, *x, w*_off-axis_, *w*_(010)_, *w*_(-110)_, *w*_(100)_, *w*_(0-11)_), 5 for the mesophase (*D,*$\;\mu_{\text{Pnc}},$ $\sigma_{\text{Pnc}}$*,* $\Delta X_{\text{Pnc}}$, *a*) and 3 for the amorphous phase (*A*, $\mu_{1}$, $\sigma_{1}$). Since the displacement values Δ*X*_12,_ Δ*X*_3_ of the Debye Waller factors have approached 0 during the initial fitting, these parameters were set to 0 for the final fit. A different orientation parameter, *p*_off-axis_, had to be applied to the peaks on the first layer line in order to improve the fit, by sharpening those reflections. *w*_off-axis_ was used as a general width parameter for all other reflections than the equatorial reflections and the (0-11) reflection.

##### 2.2.2.5 Poly-3-hydroxybutyrate monofilaments

The calibrated sample to detector distance had to slightly be decreased from 16.82 cm to 16.75 cm, so that the peak locations of the as-spun fiber matched the calculated peak positions from the unit cell, with lattice parameters: *a* = 5.73 Å, *b* = 13.15 Å and *c* (fiber axis) = 6.02 Å. Upon stretching, the *a,b* unit cell parameters had to be slightly decreased and the *c*-parameter had to be increased for a better fit; For 128 MPa: *a* = 5.72 Å, *b* = 13.12 Å, *c* = 6.03 Å. For 168 MPa: *a* = 5.70 Å, *b* = 13.08 Å, *c* = 6.05 Å. This corresponds to a total compression of the in-plane parameters by -0.5%, and an elongation of the lattice parameter along the fiber axis by +0.5%. We have fitted the crystalline and amorphous phase in the WAXD profiles [1], and have added an equatorial streak, which was simulated with a damped broad Gaussian function. 10 fitting parameters were used for the crystalline part (*p, p*_off-axis_, *p*_0_, w_off-axis_, w_(020)_, w_(110)_, $\Delta X_{12}^{crystal}$, $\Delta X_{3}^{crystal},\;f$, *k*_crys_). A second orientation parameter was used for the off-axis reflections, p_off-axis_, in order to obtain a better fit, analogously to the procedure used for PET. Additionally, we have noticed that the unoriented crystalline fraction had *f* times smaller widths than the respective equatorial reflections. The unoriented fraction has shown an elliptic trace, thus, we have introduced the parameter, *k*_crys_. Eq. (6), in the publication by Perret and Hufenus [1] was therefore changed for the unoriented fraction, to the following equation:

(3) $$\bar{I\left(s,\varphi_{hkl}\right)}=\frac{1}{\left(4\pi w{\left|s_{hkl}\right|}^{2}\right)}I_{hkl}\left(s_{hkl}\right)\frac{1}{1+{\left[\pi \left(\sqrt{s_{12}^{2}+k_{crys}s_{3}^{2}}-s_{hkl}\right)/\left(f\;w\right)\right]}^{2}}$$

5 fitting parameters were used for the mesophase (*D,*$\;\mu_{\text{Pnc}},$ $\sigma_{\text{Pnc}}$*, a,* $\Delta X_{3}$), 6 for the amorphous phase (*A, B*, $\mu_{1},\;\mu_{2}$, $\sigma_{1},\sigma_{2}$), 4 for an equatorial streak (C_,_ $\mu_{1}$, $\sigma_{1}$, ΔX_st_) and 1 parameter was used to account for the constant background arising from air scattering.

## Funding Sources

Part of this work was funded by the Swiss Innovation Agency Innosuisse (project number: 26744.1).

## Ethics Statement

The authors followed generally expected standards of ethical behavior in scientific publishing throughout article construction.

## CRediT authorship contribution statement

**Edith Perret:** Software, Formal analysis, Investigation, Data curation, Writing – original draft, Visualization. **Rudolf Hufenus:** Supervision, Project administration, Writing – review & editing.

## Declaration of Competing Interest

The authors declare that they have no known competing financial interests or personal relationships which have, or could be perceived to have, influenced the work reported in this article.
